# Access to digital finance: Equity crowdfunding across countries and platforms

**DOI:** 10.1371/journal.pone.0293292

**Published:** 2024-01-08

**Authors:** Saul Estrin, Susanna Khavul, Alexander S. Kritikos, Jonas Löher

**Affiliations:** 1 Department of Management, London School of Economics, London, United Kingdom; 2 Lucas College and Graduate School of Management, San José State University, San José, California, United States of America; 3 DIW Berlin, Berlin, Germany; 4 CEPA at University of Potsdam, IAB Nuremberg, Nuremberg, Germany; 5 GLO Essen, Independence, Virginia, United States of America; 6 Institut für Mittelstandsforschung (IfM) Bonn, Bonn, Germany; Universite Paris Pantheon-Assas, FRANCE

## Abstract

Financing entrepreneurship spurs innovation and economic growth. Digital financial platforms that crowdfund equity for entrepreneurs have emerged globally, yet they remain poorly understood. We model equity crowdfunding in terms of the relationship between the number of investors and the amount of money raised per pitch. We examine heterogeneity in the average amount raised per pitch that is associated with differences across three countries and seven platforms. Using a novel dataset of successful fundraising on the most prominent platforms in the UK, Germany, and the USA, we find the underlying relationship between the number of investors and the amount of money raised for entrepreneurs is loglinear, with a coefficient less than one and concave to the origin. We identify significant variation in the average amount invested in each pitch across countries and platforms. Our findings have implications for market actors as well as regulators who set competitive frameworks.

## Introduction

Entrepreneurial activity is an important tracker for the health of an economy. Entrepreneurial ventures are closely associated with innovation, job creation, and growth. However, they are also constrained by the supply of capital [1–5] The emergence of equity crowdfunding (ECF) as an additional source of entrepreneurial finance [6–11] opens the potential of social networks to generate funding through the engagement of new, often small-scale, private investors, referred to as “the crowd” [12, 13]. Digital mediation in this virtual capital market reduces transaction costs that previously limited the scale and efficiency of entrepreneurial financial markets [14–16]. As a result, ECF potentially increases access to external capital for innovators and entrepreneurs whose traditional investors are private angel networks, friends, and family, in the early stages, or banks and venture capitalists [17–19] in the later stages.

ECF is growing, but researchers have yet to identify how best to harness the behavior of new crowd investors to increase the supply of funds to entrepreneurial activity. In fact, most research on ECF focuses on either the choices within a single platform or the venture characteristics that lead to successful funding in the ECF environment [20]. Such research provides important insights into how to optimize individual pitches, but the generalizability of the findings is not easy to establish. Institutional and regulatory environments differ across jurisdictions [21], and the practices of ECF platforms vary considerably [22]. Evidence from diverse institutional, organizational, and regulatory contexts would be valuable to multiple stakeholders in entrepreneurial finance, especially entrepreneurs, investors, and platform originators but also policymakers who create the regulatory frameworks within which ECF financing is raised.

In this article, we propose a framework based on scaling effects to characterize the determinants of the amount of money raised from ECF investors. This framework is applied across national and organizational contexts and allows us to address our first research question: *do increases in the size of the active ECF network and*, *therefore*, *the number of investors per pitch create opportunities for more than proportionate increases in investment*? This links to an important focus of the literature on digital platforms [14, 15, 23, 24], which posits that the engagement of network participants increases more than proportionately as the network size increases [25–27]. Thus, the potential for increasing returns to social networks may exist. In contrast, in traditional entrepreneurial financial markets, high transaction costs arising from information asymmetries between two sides of the market are expected to restrict scale economies [13, 16, 19]. However, barriers are greatly reduced when, as on ECF platforms, the exchange of information is digital [14]. This implies that the virtual market for equity in entrepreneurial ventures may grow much larger and, indeed, may benefit from scaling effects where investment increases disproportionately with the size of the network. As with other networks, this would occur if increases in network size disproportionately enhanced knowledge exchange [28]. We explore the question of ECF scalability by characterizing the relationship between the number of participating investors and the amount of money raised across pitches. In our model, we aggregate across entrepreneurs on the demand side, investors on the supply side, and impose an equilibrium condition that demand equals supply to generate a locus of ECF equilibrium outcomes between the number of investors and the amount of money raised.

We posit that because of scaling effects, as platforms grow and, consequently, as the number of investors who participate in pitches increases, entrepreneurs will obtain larger amounts of funding. We use a dataset we collected from the most important ECF platforms in the UK, Germany, and the USA operating between 2011–2019 in UK and Germany and 2016–2019 in the USA. We estimate this equilibrium locus and find evidence of scalability. In fact, the relationship between the number of investors and the amount of money raised for entrepreneurs across pitches is positive and loglinear. Thus, we identify similar network effects in ECF to those observed in other digital marketplaces [14, 15, 27]. However, we also find that the relationship plots a curve concave to the origin. Hence, for ECF the “returns on the margin”, to the amounts raised, diminish along the equilibrium locus as the number of participating investors increases.

We argue that this effect occurs because the growing flow of information about investment opportunities around the network, as it expands, does not offset a decline on the margin in the amount that incremental investors are willing to supply. Therefore, we observe that as the number of investors who invest increases, the amount that they invest declines. We offer a set of explanations based on distinguishing between the types of information investors provide: soft as against hard information (16, 29, 30]. We conjecture that the observed effects in our data result from a supply-side information phenomenon whereby, as the number of investors in the pitch increases, the marginal value to the provision of soft information of each additional investor decreases.

Our second research question concerns the generalizability of these findings across diverse national and organizational contexts. We ask whether the relationship between the size of the network of participating investors and the amount invested maintains its concavity to the origin: *is there systematic heterogeneity in the ECF process arising from differences between countries or variation in the architecture of platforms*? In fact, we find the ECF equilibrium locus is positive and loglinear in each country in our sample. However, the amounts raised for a given number of investors varies by context: in particular the intercept and sometimes the slope of the relationship are sensitive to the national environment. Moreover, we also identify platform-specific heterogeneity.

Our finding of diminishing returns to incremental investment applies generally to ECF, but the precise point of inflection is sensitive to country and firm specific factors, which we conjecture arises as a consequence of institutional and organizational arrangements. We offer two explanations. First, drawing on our prior argument that soft information pooling may explain why we observe a declining marginal amount invested in pitches, differences across countries, institutional arrangements, and regulatory frameworks create heterogenous incentives for investors to contribute soft information to pitches [16, 29, 30]. Second, platforms are not all designed to maximize the impact of soft information; in fact, platform architecture prioritizes soft information provision and its interpretation in heterogeneous ways.

Our findings have important implications for a range of stakeholders including entrepreneurs, investors, platforms, and policymakers. From one perspective, the possibilities of scaling may lead entrepreneurs to pursue more ambitious and perhaps riskier strategies. From another perspective, in contrast to other digital industries, where digital agglomeration effects often lead the relationship between users and transactions to be convex, we find that the ECF market displays a concave to the origin relationship. This has important lessons for policymakers in terms of ECF market structures and regulatory environments to support them. It suggests that ECF may support relatively more competitive market structures than other digital markets because the economic benefits of scaling decline as the number of investors rises.

## Methodology

### Empirical context

Our dataset covers three major economies: the UK, Germany, and the USA. ECF is an emerging phenomenon around the world. We selected the UK, Germany, and the USA in order to capture and test the heterogeneity of institutional and regulatory environments. Institutional environments include differences in the UK, German, and US legal systems with respect to company law, securities regulation, and consumer protection, as well as the evolution of crowdfunding regulation and informal norms that emerged around this investment practice. Further, each of the three countries has a market that, although initially fragmented, has coalesced around several relatively large platforms for raising equity crowdfunding. To that end, we are able to compare equity crowdfunding across seven platforms each with different organizational origins and platform architectures. Given that most ECF studies focus on single platforms in individual countries, we believe that our focus on three major economies and seven platforms allows us to investigate whether there is systematic heterogeneity in the ECF process due to variation in organizational arrangements on platforms. Finally, the UK, Germany, and the USA differ significantly in how access to finance is sought, raised, and allocated. Our empirical focus on the number of investors that pitches attract and the amount raised per pitch describes the interaction between entrepreneurs and investors as key participants in the market for digital equity finance. To this end, we hand-collected data from the most prominent platforms in terms of volume of financing activity during their respective operational windows starting in 2011 for the UK and Germany and 2016 for the USA. That is, in the UK we focused on Crowdcube and Seedrs, in Germany, on Seedmatch and Companisto and in the US on WeFunder, StartEngine, and Republic. We briefly summarize the historical evolution of ECF in each country before turning to our model, estimation, and dataset.

#### United Kingdom

The UK venture capital market is relatively small, compared to the US, and there have been some difficulties in financing the early stages of entrepreneurship [21]. Consequently, ECF emerged in as a digital financial innovation when during the 2008–2010 banking crisis banks reduced financing for small business and exacerbated the funding gap [16]. Since around 2010, the UK has been a leader in exploiting existing regulations to permit and support equity crowdfunding as a way of raising capital for entrepreneurs. The UK regulatory environment has been more open to ECF than in much of continental Europe and the USA, though, over time, some harmonization has taken place with the passage of EU regulation and the JOBS Act in the USA. In particular, UK regulators have been willing to allow experiments with new financial technologies, using for example the ‘Regulatory Sandbox,’ providing an environment where firms are exempt from the usual regulatory consequences of engaging with Fintech innovations. Perhaps as a consequence, the number of platforms increased from four in 2010 to around 40 in 2015, though significant consolidation quickly followed the rapid entry into the equity crowdfunding market. Entry of international competitors from the USA and Europe, as well as the proliferation of niche industry specialist platforms fueled this growth. In the UK, we include Crowdcube and Seedrs which jointly represent the oldest and the largest ECF platforms both in terms of the number of investors and the amounts raised. Crowdcube started raising funds in 2011 and Seedrs shortly thereafter in 2012. During the period under observation, the two platforms had different organizational arrangements for example in terms of how individuals could participate in pitches and the governance roles that the platforms expected, but each platform also had an all-or-nothing funding model and both pursued some measure of international expansion.

#### Germany

Despite its recent growth, the German venture capital market is comparably small. In relation to the national GDP, the amounts invested are below those in the UK and the USA. Furthermore, governmental VCs play a significant role in funding innovative young firms, and regional banks run numerous publicly supported loan programs to finance young firms. In Germany, equity crowdfunding emerged in 2011 and entry of crowdfunding platforms followed. Initially, no specific regulations for equity crowdfunding were in place, and existing laws from banking, capital markets and trade regulations were applied [31]. Germany has had a traditional focus on investor protection, and starting in 2015, the Small Investor Protection Act began to regulate the German crowdfunding market. We included two German equity crowdfunding platforms, Companisto and Seedmatch: both lead the market in terms of total amounts raised and the number of successful funding rounds. Seedmatch was launched in 2011, and Companisto in 2012. Although there are differences between the organizational arrangements for funding on the platform, both platforms adhered to funding equity investments with similar upper limits and both apply the all-or-nothing funding model.

#### United States

The USA has robust venture capital, corporate venturing, and angel investor communities, all of whom contribute to the availability of capital for entrepreneurial finance. Historically, venture capital markets were geographically highly skewed, while angel financing was available through many local and regional networks. Although the banking system functions well for established ventures, except for a few specialists, US banks are not providers of capital for high-risk entrepreneurial ventures. Likewise, government funding is limited. Private investments into high-risk entrepreneurial ventures traditionally required accreditation and proof of a high level of net worth. The USA was a latecomer to equity crowdfunding, although it was a leader in rewards, donation, and debt crowdfunding. Until the passing of the Jobs Act in 2012, the broad spectrum of investors could not easily participate in the private market for entrepreneurial equity because traditional securities regulation, in place for decades, severely restricted the public sale of securities. The passing of the JOBS Act in 2012 and its subsequent implementation in 2016 unified the market for equity crowdfunding with national rather than state-based regulations, which emerged initially fill the void at the Federal level. New regulations lowered the requirements for participation in the market for private equity, created a tiered structure of amounts that firms could raise, and licensed platform intermediaries such as the three represented in our research. Like in the UK and Germany, the equity crowdfunding market in the USA saw rapid entry and then significant consolidation. As a large market, the USA has a number of platforms around which ECF has coalesced, and we include WeFunder, StartEngine, and Republic, three of the earliest and most prominent platforms.

### Model

We are interested in the scalability of the ECF process, which we characterize in terms of the relationship between the number of investors and the amount of money entrepreneurs raise in a pitch. We start with a model of the entrepreneurial capital market, in which we aggregate across entrepreneurs on the demand side, and investors on the supply side. We then impose an equilibrium (demand equals supply) condition to generate a locus of ECF outcomes between the number of investors and the amount of money raised. The resulting estimating equation allows us to address our first research question. The equation can be extended to the analysis of heterogeneity due to differences in platforms and countries, which addresses our second research question.

Our analysis is at the level of each pitch-level raise on an ECF platform. The platform enables each pitch (p), p = 1,….,n, to occur for a given time period. Pitches can be successful or unsuccessful, but in the all or nothingfunding model, actual transactions only occur in the former case, when the predetermined target for funding set by the entrepreneur is reached; otherwise, any pledges to supply funds are not taken up. Concerning the amount raised by ECF platforms for entrepreneurs, we focus solely on successful pitches where the pledges are converted into actual payments in return for shares in the venture. The platforms that are included in our research follow the all-or-nothing funding model.

To model the process, we start on the supply side. Each platform attracts a large number of individuals as potential investors. The number of potential investors on the platform is far larger than the number participating in any particular pitch and often includes individuals who have never previously participated. Thus, a large stock of potential investors stands behind the supply of funds offered in any pitch. The stock of potential investors and therefore the size of the potential investor network depends on such factors as the age and performance of the platform, the returns it offers relative to other asset classes, the costs of involvement in ECF, and the regulatory environment [16, 22]. However, a successful pitch represents a single market transaction on the ECF digital marketplace, in which the number of potential investors on the platform is narrowed down to actual investors who are then brought together with an entrepreneur seeking funds for a specific project.

We denote the total supply of funds from all interested investors in the form of equity within a successful pitch as F^s^, where F^s^ is itself a function of the stock of potential investors. We decompose the supply of funds into two parameters for each pitch: the number of actual investors in the pitch (n) and the amount invested by an investor in each pitch (*a*). Hence for each p, we propose,

$${\text{F}}^{\text{s}}=\text{n}\left(.\right)*a(.).$$ (1)

Both the number of investors and the average amount invested in each pitch may be sensitive to pitch-specific, platform-specific, and country-specific factors. For example, the number of investors and the average amount invested in a pitch may depend not just on the price of the equity offered, the investors’ evaluation of the potential future profitability of the company, and their attitude to risk, but also on the regulatory environment and specific limitations such as a minimum or maximum amount of investment. As the literature stresses [20, 32], future profitability of the entrepreneurial venture is itself associated with the nature of the business, including salability and innovativeness of the product, likely future competition, and the human capital and commitment of the entrepreneur or the entrepreneurial team.

For the demand side of each pitch, the all or nothing funding model implies that the entrepreneur presents a request for funds to investors on the platform, F^d^, which is *fixed* at the given equity price. Thus, the amount raised (A) is pre-determined,

$${\text{F}}^{\text{d}}=\text{A}.$$ (2)

The virtual market reaches equilibrium for each pitch when demand equals supply,

$${\text{F}}^{\text{s}}={\text{F}}^{\text{d}}.$$ (3)

In fact, on some platforms there may be overfunding, which implies that F^s^> = F^d^. However, for these platforms, equity prices are adjusted *ex-post* so that demand and supply of funds are brought into equality and Eq (3) still holds exactly. We tested for the impact of whether the pitch was overfunded. Since the variable was not significant and did not affect the other parameters of interest, we do not pursue this line of enquiry.

Our empirical analysis estimates the locus tracked by A and n across pitches. To derive this, we substitute Eqs (1) and (2) into (3), rearrange terms, and subscripting by p for each pitch, derive the equation:

$${\text{A}}_{\text{p}}={\text{n}}_{\text{p}}\left(.\right)*a_{\text{p}}\left(.\right)$$ (4)

which we estimate across pitches p = 1,…,n.

Eq (4) is the basis of our estimating equations. As noted above, both *a* and n may vary across time and may be sensitive to the location (L) and sector (S) of the new venture as well as possible institutional and regulatory effects, which we capture through the use of country-fixed effects and variables denoting particular ECF platforms.

### Dataset

We collected data on completed fundraising pitches from each platform’s publicly-facing websites from when the platforms started operation until the end of our observation windows in 2019. This yielded raw data on 2,189 pitches. After checking the data, we dropped observations if they did not have complete information on the number of investors, location, or sector. Our dataset contains 1,872 observations of successful pitch level raises. After accounting for incomplete data, there are 228 pitches in Germany (DE), 1,295 in the UK, and 349 in the US. In the observation window, the two main platforms in Germany–Seedmatch and Companisto–had 124 and 104 pitches respectively; in the UK, Crowdcube and Seedrs had 674 and 621 pitches respectively; and in the USA, WeFunder had 135 pitches, Republic 56 pitches, and StartEngine 158 pitches. The amounts raised for new ventures in our dataset during the period we analyzed is over $1.05 billion, a considerable amount for early venture finance. Across the seven platforms, the average amount raised per pitch was slightly over half a million USD. It should be noted that for all platforms we collected information through public websites. We made full effort to confirm the information, for example in the USA through SEC filings, but it may be the case that not every successful pitch is reflected in the dataset and not every pitch had full information that we needed for variables in the regression.

### Variable definitions

The main outcome variable is the total amount raised per pitch and the primary independent variables are the number of investors in each pitch. In some specifications, to address potential issues of non-normality, one or both values are log transformed into lnA and lnN respectively. In addition, we include country and platform dummies to test for heterogeneity across institutional environments and platform architecture. We included a number of control variables as fixed effects. Specifically, the year in which firms raised capital on the platforms, classification of each firm by industry for which we used the UK SIC codes, and regional classification of NUTS3 in UK and Germany, and CBSA in the USA.

### Estimation

We estimated three OLS regression models with log-log specifications in our analysis and tested these with and without fixed effects.

## Results

Table 1 reports estimates of the model, Eq (4), with standard errors clustered by firm. We estimated linear, quadratic semi-log, and quadratic loglinear specifications as well as the loglinear one that is reported and chose the reported specification based on goodness of fit. The results are quite similar but the R-square of 0.64 in the loglinear specification with fixed effects is the highest. Our estimates both exclude (column 1) and include (column 2) sector, locational, and time fixed effects. The FE are highly significant and raise the R-squared by 0.19, highlighting the important role of regional and sectoral heterogeneity in the ECF funding process. The equilibrium locus is found to be exponential, but the estimated coefficient is significantly less than unity. Hence, the underlying equation is concave to the origin. The estimated coefficient in the fixed effect model (column 2), which provides our best estimate of the elasticity of investment to the number of investors is 0.75.

**Table 1:** Relationship between amount raised and number of investors.

VARIABLES	(1)	(2)
	lnA	lnA
lnN	0.81313***	0.74690***
	(0.02027)	(0.02401)
Constant	8.17842***	5.80269***
	(0.10735)	(1.59521)
Observations	1,872	1,872
R-squared	0.46257	0.63573
SIC FE	No	Yes
Year FE	No	Yes
Nuts FE	No	Yes

Standard errors in parentheses

*** p<0.01,

** p<0.05,

* p<0.1

**Fig 1:**
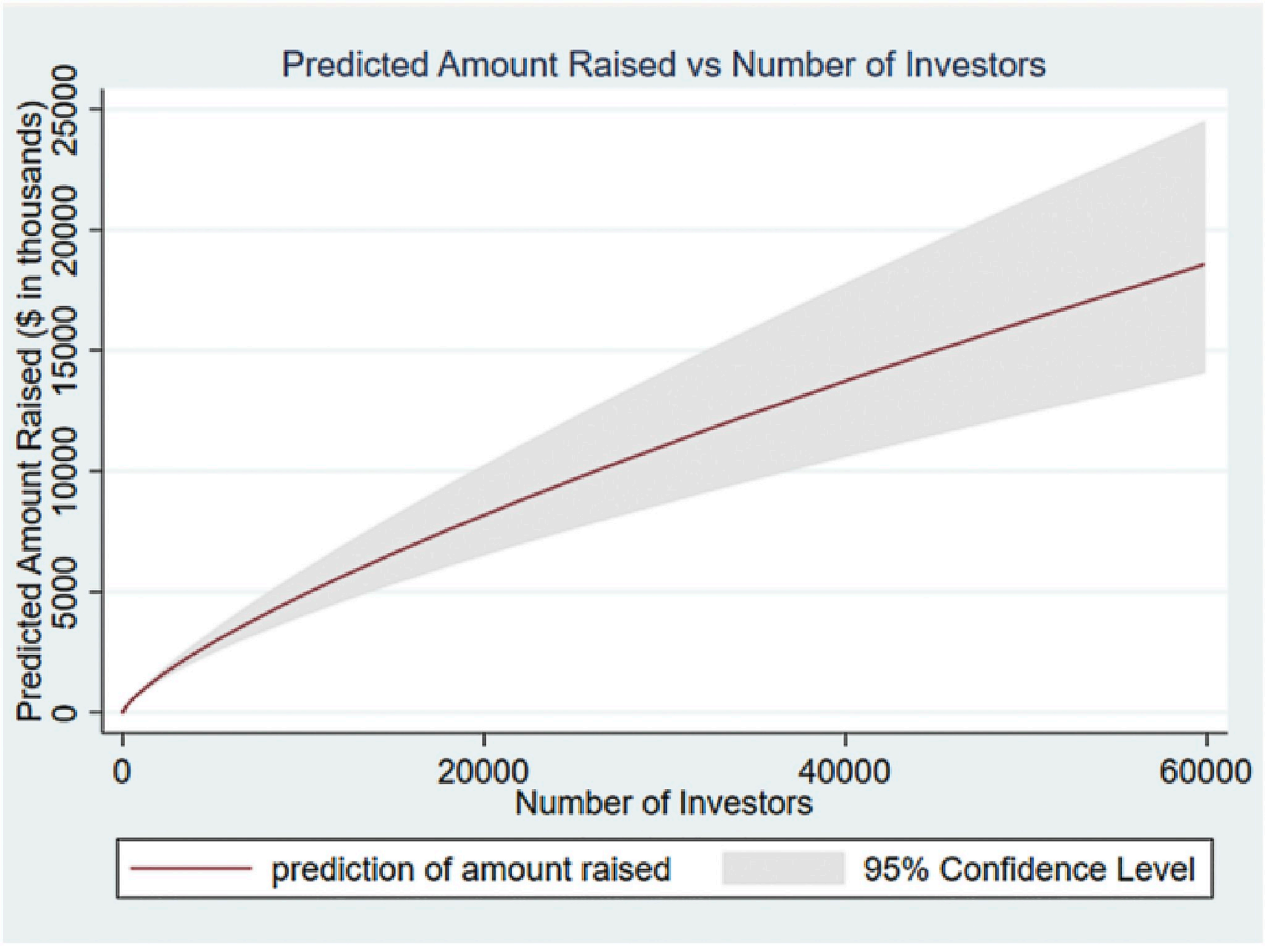
Relationship between predicted amount raised and number of investors.

In Fig 1, we plot the locus of equilibria across pitches reported in column 2 showing the actual amounts raised and the number of investors on the two axes. Fig 1 reveals that the specification has a precise fit with narrow confidence intervals. We note that there are three outlier values in the data in terms of the number of investors and the amount raised. These have been excluded from Fig 1 for presentational reasons. However, they are included in the analysis and the results are not materially affected if Models 1 and 2 of Table 1 are re-estimated excluding these outliers and by winsorizing the dataset by 2.5%. We retained the outliers in the analysis for regressions in Tables 2 and 3 where results are also not affected.

**Table 2:** Relationship between amount raised, number of investors, and country effects.

VARIABLES	(1)	(2)	(3)	(4)
	lnA	lnA	lnA	lnA
lnN	0.82262***	0.77358***	0.94234***	0.95217***
	(0.02831)	(0.03054)	(0.03115)	(0.03316)
DE	0.35655***	0.64655***	-0.12468	0.83476**
	(0.05727)	(0.08536)	(0.30600)	(0.35390)
UK	0.90622***	1.07531***	1.72524***	2.28415***
	(0.04630)	(0.06443)	(0.25695)	(0.29764)
lnN_DE			0.06115	-0.05509
			(0.05560)	(0.06063)
lnN_UK			-0.16412***	-0.23786***
			(0.04769)	(0.05239)
Constant	7.45896***	8.52210***	6.86667***	5.88859***
	(0.14437)	(0.16861)	(0.16000)	(0.22182)
Observations	1,872	1,872	1,872	1,872
R-squared	0.53805	0.57674	0.54205	0.58242
SIC FE	No	Yes	No	Yes
Year FE	No	Yes	No	Yes
Nuts FE	No	No	No	No
Cluster	Firm	Firm	Firm	Firm

Robust standard errors in parentheses

*** p<0.01,

** p<0.05,

* p<0.1

**Table 3:** Relationship between amount raised, number of investors, and platform fixed effects.

VARIABLES	(1)	(2)	(3)	(4)	(5)	(6)
	lnA	lnA	lnA	lnA	lnA	lnA
lnN	0.77250***	0.69687***	1.25545***	1.14663***	0.95365***	0.93028***
	(0.03514)	(0.04535)	(0.06488)	(0.08215)	(0.03487)	(0.05590)
Crowdcube	0.06259	0.17331				
	(0.05837)	(0.14536)				
Companisto			-0.74014***	-0.85201***		
			(0.09001)	(0.11261)		
StartEngine					0.38628***	0.48343***
					(0.08656)	(0.15981)
WeFunder					0.63983***	0.58969***
					(0.07482)	(0.13882)
Constant	8.58851***	7.63892***	5.58411***	6.74277***	6.38832***	6.70155***
	(0.20169)	(0.28028)	(0.36496)	(0.57082)	(0.20522)	(0.23909)
Observations	1,295	1,295	228	228	349	349
R-squared	0.42806	0.54896	0.73791	0.91911	0.78107	0.87661
Country	UK	UK	DE	DE	USA	USA
SIC FE	No	Yes	No	Yes	No	Yes
Year FE	No	Yes	No	Yes	No	Yes
Nuts FE	No	Yes	No	Yes	No	Yes
Cluster	Firm	Firm	Firm	Firm	Firm	Firm

Robust standard errors in parentheses

*** p<0.01,

** p<0.05,

* p<0.1

### Differences by country

We use the model in Eq (4) to consider other factors that might affect ECF platform behavior across countries. The entrepreneurial finance landscape, the regulatory environment, and the history of equity crowdfunding differ considerably between the countries in our sample [13, 21, 22, 33]. These lead us to expect that the UK, with its early start and a benign regulatory environment, could display a higher average funding per investor, compared with Germany, which initially regulated ECF more vigorously in order to protect investors. The USA is a much larger market but has been slower to develop ECF and has better-developed alternative funding sources, such as angel investing and venture capital.

We re-estimate the baseline model using a variety of specifications of country dummy variables, with the results reported in Table 2. USA is the omitted country. In unreported regressions, we also show that the quadratic specification shown in Table 2 continues to be slightly preferred to the linear and semi-log specifications using the criterion of goodness of fit. Columns 1 and 2 report the results by assuming that country-specific heterogeneity affects the intercept of the loglinear equation but not the slope: only country-specific dummies are included. In columns 3 and 4, we allow for effects to impact both the slope and the intercept of the model. Because of the overlap between country and regional fixed effects (all the regions taken together capture all the locations in a given country), we exclude the regional controls in these models. Paralleling previous tables, Columns 1 and 3 exclude all fixed effects, while columns 2 and 4 include the fixed effects for sector and year.

Comparing the regressions in columns 1 and 2, and 3 and 4 and relative to the USA, the locus is everywhere higher in Germany, but the slope is not significantly different: the interaction term between the number of investors and the Germany dummy (lnN*DE) is not statistically significant. Thus, the differences between the USA and Germany are significantly explained by a shift in the intercept; the (loglinear) locus is significantly higher in the latter. Thus, holding all other factors constant, the average amount invested in each pitch is higher in Germany than in the US.

The comparison between the UK and the other two countries is more nuanced because both the slope and the intercept are significantly different in the UK from the USA, see Table 2 columns 3 and 4. Relative to the USA, we see from Table 2, column 4 –the best fitting regression–that the intercept of the locus is higher in the UK and that the slope is also initially higher but declines more quickly with lnN. As a result, the loci for UK and USA cross: the average amount invested in each pitch is higher in the UK than in the US when the number of investors is small, but lower when the number of investors is large.

To compare the UK and Germany, in Table 2, we see that the locus for the latter lies initially below the UK when the number of investors is small. However, the slope declines more slowly as lnN increases so at some point the locus for Germany crosses that of the UK.

A simple way to obtain comparability in the effect across the three countries is to make the (strong) assumption that the country-fixed effects impact only the intercept but not the slope of the equation. This is reported in Table 2, column 2. In this model, the upward shift in the line relative to the USA is most marked in the UK, then Germany. Thus, the intercept in the loglinear locus increases from 8.52 in the US to 9.12 in Germany and to 9.58 in the UK. In Germany and the UK, a given number of ECF investors provide a larger average amount of funds to entrepreneurs than the equivalent in the USA. Thus, Table 2 supports the view that institutional variation across jurisdictions significantly affects the fundraising performance of ECF platforms. Given the differences in the regulatory environment and the ECF history, the observed pattern is consistent with prior expectations.

### Differences by platform

Finally, organizational differences across the ECF platforms may represent an additional source of heterogeneity in equity fundraising performance. For example, in the UK, during the period we study, Crowdcube and Seedrs differ with respect to ownership and governance relationships for the shares that have been purchased [21, 34] while, in Germany, the minimum investment threshold differs between Seedmatch and Companisto. To explore this issue, we estimate our baseline model for each country separately but additionally include platform specific dummies. Thus, in Table 3 we report regressions estimated on the three country sub-samples: UK in columns 1 and 2; Germany in columns 3 and 4; and USA in columns 5 and 6. Note that this approach leads each equation to have a smaller sample size. Paralleling Table 1, the first, third, and fifth columns are estimated without sectoral, regional, and time fixed effects, while the second, fourth, and sixth columns include the relevant fixed effects. The omitted category is Crowdcube in the UK, Seedmatch in Germany, and Republic in the USA. Further, since Table 3 is estimated at the country level, we include regional fixed effects.

We find support in Table 3 for the view that there is platform-specific heterogeneity in ECF performance, at least in the USA and Germany. Thus, we find that pitches on the Companisto platform yield a significantly lower average investment than for Seedmatch, while in the USA, both StartEngine and WeFunder display higher average investment rates per pitch than Republic. These differences are quite large: the constant in the performance equation is lower by about 12.6% in Companisto than Seedmatch; and higher by around 4.6% in both StartEngine and WeFunder relative to Republic. The coefficient on lnN is different between the three countries but we do not focus on these results to explain cross-country variation because of the small sample size. However, the average amount raised per investor by Seedrs is not significantly different to that of Crowdcube.

## Discussion, implications, and future directions

In this paper, we address two research questions. The first asks whether an increase in the number of participating investors in a pitch creates a more than proportionate increase in investment, while the second questions if systematic heterogeneity in ECF that arises from institutional differences across countries or from organizational differences across platforms. To answer these questions, we created a large dataset that included successful financing activity on seven platforms operating in three countries (UK: Crowdcube and Seedrs; Germany: Companisto and Seedmatch; and USA: WeFunder, StartEngine, and Republic). We model the ECF digital market equilibrium and derive a locus of equilibria between the number of investors and the amount invested by pitch. This framework allows us to analyze the scaling of ECF as digital finance in a way that captures different institutional (country) and organizational (platform) contexts.

We find that average investments per pitch change with the number of investors in a loglinear function with an elasticity of less than one—in fact around 0.75—so the equilibrium locus between the amount invested and the number of investors is concave to the origin. This implies that the marginal amount invested in ECF declines as the number of investors increases. In contrast to the pattern observed in some two-sided networks, the returns on the margin to the amounts raised in our analysis diminishes along the equilibrium locus as the number of investors increases.

The literature on scale effects in networks might lead one to expect that the coefficient would be greater than one because opportunities for the exchange of information expand more than proportionately as the network size increases [14]. However, the network effects appear to be more nuanced when we consider the network of actual investments on ECF platforms where the marginal amount raised from additional investors in the pitch actually declines. There are several potential explanations. If incremental investors are less well-resourced than those who participate in ECF pitches when the number of investors in a pitch is smaller, then this could also explain the concave shape of the locus. This reflects diminishing resources. Another explanation invites us to consider ways in which the expansion of the size of pitch may be bringing in additional investors with less valuable information. Thus, while the possibilities for mutually beneficial information exchanges increase as the overall network expands, the benefits gained from those exchanges decline as the number of investors in a pitch increases.

To understand why benefits from information exchange may decline at the margin, we build on the distinction between soft and hard information. Theoretically, this distinction originates in the information perspective and has been used in the finance literature. It offers one explanation of how the character of information in the ECF process affects investment decisions [16, 29, 30]. Hard information is defined as facts about which there is general agreement, which are verifiable, and do not change in the relatively short duration of the pitch during which investors make their decisions [16, 29]—for instance, the entrepreneur’s education and skills or the firm’s age, and size. In contrast, soft information, for example the firm’s growth potential, is open to debate and is more difficult to verify [16, 29]. In principle, ECF platforms play a significant role in reducing the transactions costs of exchanging both hard and soft information in the investment process. ECF platforms allow information exchange to scale. Increases in the size of the ECF overall network increase the quantity of information. Consequently, the number of investors in each pitch will also increase the amount of soft information circulating around the platform. We conjecture that incremental sense-making about soft information is subject to diminishing returns. This is because soft information is difficult to verify and as a larger number of interpretations about it are expressed the quantity of useful additional information concerning an investment may not increase in proportion. Distinguishing between hard and soft information opens the door for future conceptual explanations and empirical testing about the role of different types of information on platforms [16].

### Implications for stakeholders

In summary, the overall shape of the relationship between the amount invested and the number of investors is stable, but we find significant heterogeneity across countries and platforms. We propose this finding to be a result of variation in the institutional and regulatory arrangements across countries as well as organizational arrangements across platforms. Our empirical results underscore the importance of moving towards multi-country and multi-platform datasets. Recent research has started investigating differences across platforms [35, 36]. This stream of research identifies substantial variation between and across platforms in the rights of investors, requirements for co-investing, and in the selection process of entrepreneurs [20]. In addition, comparisons across multiple countries documents variation across crowdfunding models [33]. Finally, research has shown that quality of regulation, financial development, and the reach of digital access affect the volume of crowdfunding activity [37]. Our work contributes to this emerging stream of research, and we now offer some generalizable implications that are potentially valuable to multiple stakeholders in entrepreneurial finance including entrepreneurs, investors, platforms, and regulators, the latter of whom often struggle to create competitive frameworks for emerging innovations.

#### Implications for entrepreneurs

ECF platforms harness significant capital across countries and platforms. As a new digital source of finance, ECF is an additional strategic option for entrepreneurs to access capital. Indeed, as a digital marketplace based around social networks ECF is able to scale and loosen the constraints of traditional entrepreneurial investors such as angels, venture capital, or corporate venturing. Over time, the digital approach to finance, that ECF offers has the potential to capture a growing share of the entrepreneurial capital market. Moreover, ECF is mobilizing an increasing number of investors to purchase equity in illiquid and risky new ventures. As a financial innovation, ECF may help to address the funding gaps that have traditionally bedeviled entrepreneurs as their businesses grow and expand. Yet, ECF platforms vary widely with respect to requirements to join the network, the costs and terms of participation, and the ownership arrangements that pertain to successful pitches. Thus, a clear implication for entrepreneurs is to know how the platform they select manages its dual networks of entrepreneurs and investors and the extent to which the venture would benefit from its organizational arrangements in both the short and long terms. In addition, platforms often require entrepreneurs to bring their own crowd of supporters to the pitch but hope that investors already on the platform would invest as well. Our research shows that potentially there is a declining marginal benefit from securing additional investors as the number of investors in a pitch increases. Tying investors to the pitch and keeping them engaged is costly, so investor acquisition requires careful communication not only at the time of the pitch but over the life of the venture. By implication, how entrepreneurs manage the hard but especially the soft information about their ventures will shape the sense-making process investors pursue as they formulate current and future investment decisions. Further, as platforms evolve, they often seek larger raises and that include institutional investors joining alongside the crowd. This implies that entrepreneurs may confront an even more diverse set of investors, potentially more complex instruments, and likely stiffer terms. Finally, access to larger supplies of capital may lead entrepreneurs to pursue increasingly ambitious goals, especially as they move through multiple rounds of funding. The implication for entrepreneurs accessing digital finance on ECF platforms is the need to understand their investors maintain and to focus on the strategic direction of the firm and not only fundraising.

#### Implication for investors

Especially for investors, not all the implications of a fully scaled ECF sector have been ironed out. Corporate governance was an immediate concern of many investors in ECF. If an increasing number of investors finance larger demands for capital not only will ownership be dispersed but also a negative relationship between the ambition of the entrepreneur and the quality of governance *ex-post* can arise. Our research shows a declining marginal contribution to the amount raised in a pitch from additional investors. However, entrepreneurs and platforms push for larger raises and a wider set of investors, so the potential for principal-principal conflict emerges between investors with large contributions and those with investment contributions below the margin. Likewise, there is potential for similar conflicts between individual investors and institutional investors who come to join in the raise alongside the crowd. Investors need to understand the implications of different governance structures and urge platforms and regulatory authorities to guard against unwarranted dilution as well as to advocate for the protection of minority rights. As ECF evolved, regulation and organizational norms have sought to resolve these conflicts through nominee structures and other governance tools. Investors also face implications from potential for outright fraud, which in reality has been relatively infrequent, and sheer mismanagement of the ventures. We have argued that the digital architecture of ECF platforms lowers the transaction costs of gathering, interpreting, and distributing information and that this is particularly important for soft information, which allows investors to make sense of uncertainty about the venture raising money. The asymmetry of information between investors and entrepreneurs may be reduced, but it is certainly still present. Soft information requires ongoing reexamination; thus, by implication, investors should screen platforms and ventures for effective governance and communication during the pitch and insist on it after. Finally, our research showed heterogeneity across both platforms and countries. As platforms internationalize and both entrepreneurs and investors can raise money and invest across institutional boundaries, the implication for investors is to increase due diligence in the face of reduced transaction costs of international investing in a digital environment.

#### Implications for platforms

Our findings may help platforms think about suitable growth paths. It is clearly important for platforms to develop strategies that prevent or limit the decline in average investments per pitch as investor numbers grow. This explains why platforms seek upwards integration with business angel networks, venture capitalists, and offer incentives to attract wealthy investors. Likewise, bring institutional investors alongside the crowd aims to increase confidence in the pitch, which increases not only the number of investors participating but also the size of their investments. Our research indicates that the key issue for platforms is to improve the quality of their investor networks as they expand. In this context, it is important to note that if entrepreneurs bring their own crowds to pitches, then the cost of acquiring potential investors on the platform appears to be quite low. However, platforms face the on-going cost of building traction with existing investors. Specifically, when crowd investors join and make below marginal level contributions to the amount raised and then become the dormant majority of the investor network, not invest again, the platform faces the cost of continuously chasing additional active investors. Platforms are not only transaction intermediaries but also curators of the network experience on and off the digital-scape they create. Our work points to heterogeneity at the platform level within and across countries. Thus, the implication for platforms is that in order to enhance their competitive position, platforms have to balance the demand of entrepreneurs and investors on their two-sided networks. They need to create meaning to network participation that over the long-term increases value of every potential investor who joins generating same-side and cross-platform network effects. Finally, many platforms are expanding internationally in search of network expansion and diversification. In fact, recently Republic, one of the US platforms in our study bought Seedrs, one of the UK platforms included in this study. Among many other reasons, Republic purchased the regulatory rights that Seedrs has in the UK and Europe. This is a typical motivation for entry through acquisition found across industries and geographies. As ECF enters its post emergence era, international expansion either through acquisition, strategic alliances, or *de novo* geographic expansion have clear implications for platforms. Our findings suggest that even in a digital environment, institutional and regulatory differences across countries have significant effects on pitch outcomes. When regulatory regimes are increasingly harmonized, as they are in Europe, they do not erase country-level differences in institutions and norms that make scaling ECF a challenging proposition.

#### Implications for financial regulators

Our results suggest that ECF funding outcomes are higher in countries where crowdfunding regulations have been light-touch, as in the UK, where the practice evolved and became an institutionalized part of the financial ecosystem. Therefore, policymakers in other countries who aim to expand the financing choices that entrepreneurs have may use the UK’s regulatory environment as a case study [8, 16, 21]. Designing the regulatory frameworks for ECF platforms and other forms of digital finance in a way that makes them more attractive to entrepreneurs and investors will allow platforms to scale more easily as well as encourage domestic and international entry and competition. Finally, our results also indicate some important factors to be considered by ECF regulators. Until recently, in many digital industries, regulators tended to regard the potential benefits to innovation and growth as outweighing the costs in terms of consumer welfare. Hence, they have been willing, for example, to countenance large market shares in the hands of tech giants [38]. Now, calls to break the huge digital firms up in order to address dangers of monopolistic abuse are increasing. Such market power tends to be a consequence of agglomeration effects in scaling. However, our findings show that the ECF market may work differently. We find that the ECF market displays a concave to the origin relationship between supply and demand for capital. This suggests that ECF may be able to support more competitive market structures than some other network industries because the benefits to scaling decline as the number of investors rise. It indicates that the sector may merit more benevolent attention from regulators who are concerned with platforms abusing market power.

### Limitations

Our research is not without limitations. To begin, we focus on the analysis of successful pitches and do not address whether these lead to successful firms. We elected to include a parsimonious set of explanatory variables—that is, the amount raised as a function of the number of investors across countries and platforms, and controls for industry, regional geography, and time. Numerous other studies offer predictive models to explain what determines successful versus failed pitches, albeit most are single-platform or single-country studies. Likewise, an increasing number of studies focus on the post-funding performance of firms that crowdfund. Ours is a multi-country and multi-platform study that is intentional in its focus, but future studies should include variables at the level of the entrepreneur, the firm, and the competitive environment. Moreover, information about investors, the timing of their investments, and the investment amounts would further clarify the causes of the diminishing marginal returns that we find are associated with an increasing number of investors in a pitch. Likewise, the countries we consider in our analysis differ with respect to their institutional and regulatory environments for finance and attitudes towards entrepreneurship. Nevertheless, our analysis is restricted to three large developed Western economies, so the generalizability of our results would need to be confirmed not only in other emerging markets or developing economies but also other developed economies. Finally, our study captured the emerging, early phase of the ECF market in three countries, especially in the USA, and across seven leading platforms. Our findings should be confirmed in later stages of the evolution of the ECF market, which saw significant scaling-up of investment activity, as regulations changed over time, across both new entrants and incumbents, and in countries with more or less stringent investment norms.

### Future directions

Our research results point to multiple avenues for future research. Here we propose a hand-full as a call to action. We found that increasing number of investors in the pitch has a diminishing marginal contribution to the amount raised. How can platforms and entrepreneurs design behavioral interventions and offer economic incentives to raise investment amounts as pitches attract more investors? We conjecture and show that institutional and regulatory differences at the country level and organizational differences at the platform level may influence the behavior of investors. Although we offered explanations, future research should offer and test mechanisms to explain what drives the differences we observe. We would strongly encourage future work that theoretically explains and empirically shows how a wide range of institutional and organizational differences may affect the flow of soft information between entrepreneurs and potential investors and between investors. More research is also needed to identify the causes and consequences of different organizational designs at the platform level. We propose that such research address the incentives in the architecture of platforms and their digital landscapes to provide and interpret soft information. Moreover, with the implementation of more sophisticated artificial intelligence tools, would human and crowd-based sense-making of soft information become more or less valuable and prove more or less accurate? Will the introduction AI tools lead to further hardening of soft information and increased reduction of transaction costs in the scaling equity investments in digital finance? Finally, will the future of ECF be frictionless, international, and free of platform boundaries?
